# Preclinical Photodynamic Therapy Targeting Blood Vessels with AGuIX^®^ Theranostic Nanoparticles

**DOI:** 10.3390/cancers16233924

**Published:** 2024-11-23

**Authors:** Ewa Kowolik, Dariusz Szczygieł, Małgorzata Szczygieł, Agnieszka Drzał, Kalyani Vemuri, Anna-Karin Olsson, Arjan W. Griffioen, Patrycja Nowak-Sliwinska, Agnieszka Wolnicka-Glubisz, Martyna Elas

**Affiliations:** 1 Department of Biophysics and Cancer Biology, Faculty of Biochemistry, Biophysics and Biotechnology, Jagiellonian University, 7 Gronostajowa Street, 31-387 Krakow, Poland; ewa.kusber@gmail.com (E.K.); dariusz.szczygiel@uj.edu.pl (D.S.); gosia.szczygiel@uj.edu.pl (M.S.); agnieszka.drzal@uj.edu.pl (A.D.); a.wolnicka-glubisz@uj.edu.pl (A.W.-G.); 2 Department of Medical Biochemistry and Microbiology, Biomedical Center, Uppsala University, Husargatan 3, SE-75123 Uppsala, Sweden; kalyani.vemuri@imbim.uu.se (K.V.); anna-karin.olsson@imbim.uu.se (A.-K.O.); 3 Angiogenesis Laboratory, Department of Medical Oncology, Amsterdam UMC, Cancer Center Amsterdam, 1081 HV Amsterdam, The Netherlands; a.griffioen@amsterdamumc.nl; 4 School of Pharmaceutical Sciences, University of Geneva, 1211 Geneva, Switzerland; 5 Institute of Pharmaceutical Sciences of Western Switzerland, University of Geneva, 1211 Geneva, Switzerland; 6 Translational Research Center in Oncohaematology, 1211 Geneva, Switzerland

**Keywords:** photodynamic therapy, AGuIX, glioma, glioblastoma multiforme, CAM, EPR oximetry

## Abstract

**Background:** Glioblastoma multiforme (GBM) is the most common highly aggressive, primary malignant brain tumor in adults. Current experimental strategies include photodynamic therapy (PDT) and new drug delivery technologies such as nanoparticles, which could play a key role in the treatment, diagnosis, and imaging of brain tumors. **Objectives:** The purpose of this study was to test the efficacy of PDT using AGuIX-TPP, a polysiloxane-based nanoparticle (AGuIX) that contains TPP (5,10,15,20-tetraphenyl-21H,23H-porphine), in biological models of glioblastoma multiforme and to investigate the vascular mechanisms of action at multiple complexity levels. **Methods:** PDT effects were studied in monolayer and spheroid cell culture, as well as tumors in chicken chorioallantoic membranes (CAMs) and in mice were studied. **Results**: Treatment was effective in both endothelial ECRF and glioma U87 cells, as well as in the inhibition of growth of the glioma spheroids. PDT using AGuIX-TPP inhibited U87 tumors growing in CAM and destroyed their vascularization. The U87 tumors were also grown in nude mice. Their vascular network, as well as oxygen partial pressure, were assessed using ultrasound and EPR oximetry. The treatment damaged tumor vessels and slightly decreased oxygen levels. **Conclusions**: PDT with AGuIX-TPP was effective against glioma cells, spheroids, and tumors; however, in mice, its efficacy appeared to be strongly related to the presence of blood vessels in the tumor before the treatment.

## Simple Summary

Glioblastoma multiforme is the most common primary malignant brain tumor and is usually fatal shortly after diagnosis due to its rapid growth and aggressive nature. Improved drug delivery technologies such as nanoparticles are currently under intensive experimental investigation. The integration of nanoparticles with photodynamic therapy (PDT) presents a promising adjunctive treatment option which, when combined with standard therapies, may enhance the treatment of glioblastoma multiforme. This study explores the potential of PDT using AGuIX-TPP, a polysiloxane-based nanoparticle (AGuIX) containing TPP (5,10,15,20-tetraphenyl-21H,23H-porphine), as a viable candidate for this purpose. We conducted investigations using glioblastoma multiforme cells to examine the mechanisms of action across multiple levels of complexity within biological models of glioblastoma multiforme, employing spheroids, chicken chorioallantoic membranes (CAMs), and mouse models.

## 1. Introduction

Glioblastoma multiforme (GBM) is the most common primary malignant brain tumor in adults [1]. This type of tumor is usually fatal shortly after diagnosis. Dismal median survival is about 12 to 18 months after diagnosis, with an overall 5-year survival no greater than 35% [1,2,3]. GBM may develop in individuals of any age, although it is more commonly diagnosed in older adults. Developing tumors can cause worsening headaches, nausea, vomiting, seizures, and changes in behavior [1,4]. Due to the location of the tumor, diagnosis requires rather advanced methods. Diagnostic tools include computer tomography (CT) and magnetic resonance imaging (MRI). The result is then often confirmed by biopsy. First-line treatment for primary brain tumors starts with surgical resection that is largely dependent on tumor location, surgeon experience [2,3], and the use of preoperative and intraoperative techniques. Surgical resection is then complemented by radiation therapy and chemotherapy with temozolomide, a chemotherapeutic drug that damages cellular DNA. The precision of intraoperative techniques in tumor diagnosis has advanced the field of tumor surgery. Microscopy with fluorescence-based methods facilitates the spatial recognition of tissue composition, and laser interstitial therapy can be an additional treatment method [5]. It is very important to accurately diagnose the type of cancer, involving histological diagnosis, stage of lesions, or tumor biopsy genotyping, to obtain information on its molecular features. A precise diagnosis can improve one’s quality of life and increase survival [3]. The systemic therapies used in the treatment of glioblastoma are known to have side effects such as general weakness, hair loss, and persistent nausea [2]. Some issues such as resistant cancer cells, recurrence, and metastases have not been overcome, and the average survival of patients with GBM remains disappointingly short. The traditional approach typically combines surgery, radiation, and chemotherapy, particularly using temozolomide, and aims to extend one’s lifespan rather than provide a complete cure. For this reason, new technologies are essential for future cancer treatments. Current experimental strategies include immunotherapy, targeted therapy, photodynamic therapy, gene therapy, and novel drug delivery technologies such as nanoparticles, which can play a key role in the treatment, diagnosis, and imaging of brain tumors [3,6,7,8]. Anti-angiogenic approaches were also tested as adjuvant therapy [9].

Photodynamic therapy (PDT) is a treatment based on the generation of reactive oxygen species (ROS) by a compound, excited by light, in the presence of oxygen, leading to the death of cancer cells. In addition to treating cancerous lesions, PDT is also used to treat other diseases resulting from neovascularization [10], atherosclerosis [11], and restenosis after angioplasty [12], as well as arthritis, where the mechanism is based on the closure of blood vessels [13]. Moreover, this method can be used for diagnostics, especially for invisible lesions [14]. However, many factors influence the efficacy of therapy, and not every light-absorbing compound is a good drug for use in PDT, as described in a review [14]. One feature is the ability of the photosensitizer to have a selective affinity for tumor cells. With the use of targeted nanoparticles, many studies have shown improved therapeutic outcomes of PDT. Despite promising results from studies on PDT powered by nanocarriers, critical questions remain regarding the success of PDT in clinics.

Porphyrins are well-known photosensitizing agents used in the therapy of malignant tumors [15,16,17,18,19,20,21,22,23]. The synthetic compound meso-tetraphenylporphyrin (TPP; 5,10,15,20-tetraphenyl-21H,23H-porphine) is the simplest aryl derivative of the porphyrin ring system. Since TPP is not soluble in aqueous solutions, it must first be incorporated into a carrier. The effect of PDT with TPP was described for nanoparticles based on lactic acid [24], pegylated nanoparticles [25], or liposomal nano-formulations [26,27,28].

**Figure 1:**
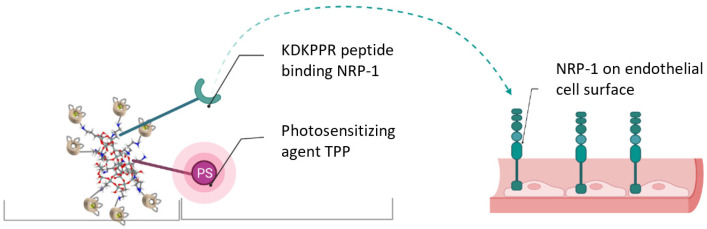
Scheme of AGuIX-TPP nanoparticles targeting vascular endothelial cells. AGuIX contains a KDKPPR peptide that binds to NRP-1 (created in BiorendeR).

In the present work, we used TPP-engrafted AGuIX nanoparticles. AGuIX-TPP is a polysiloxane-based nanoplatform (AGuIX, Activation and Guidance of Irradiation by X-ray) that combines a photosensitizer (PS); the magnetic resonance imaging (MRI) contrast agent gadolinium (Gd); and a peptide ligand motif (KDKPPR) targeting neuropilin-1 (NRP-1), a receptor overexpressed by vascular tumor endothelial cells and glioblastoma cells (Figure 1) [29]. Due to their therapeutic and diagnostic moieties, they are considered theranostic. They exhibit high longitudinal relaxivity and prolonged blood circulation, leading to a superior MRI contrast [30]. AGuIX nanoparticles have been successfully biofunctionalized with peptides and monoclonal antibodies, improving tumor-targeting efficiency [31]. In liver cancer models, AGuIX nanoparticles labeled with ^64^Cu have shown high tumor accumulation and a reduced ^18^F-FDG uptake, proving their potential as theranostic agents for image-guided radiotherapy [32]. The accumulation and retention of AGuIX nanoparticles at tumor sites have been successfully translated from preclinical models to patients, showcasing their potential based on preclinical evidence [33]. AGuIX nanoparticles with different ligands have been employed for both radiotherapy and photodynamic therapy in preclinical models [29,34,35].

As glioblastoma is considered a hypervascularized tumor, targeting the vasculature along with tumor cells might be a good strategy. We hypothesized that the nanoparticle targeted to neuropilin-1 and combined with a photosensitizer of high efficiency of singlet oxygen generation would be effective against endothelial and glioblastoma cells leading to tumor damage. Therefore, we tested the efficacy of PDT using AGuIX nanoparticles functionalized with a TPP photosensitizer and a peptide designed to target the NRP-1 receptor, overexpressed in tumor-associated vascular endothelial cells in models of glioblastoma multiforme. We demonstrate the photodynamic efficacy of AGuIX-TPP, a carrier of AGuIX combined with TPP 5-(4-carboxyphenyl)-10,15,20-tetraphenylporphyrin, in biological models of glioblastoma multiforme using spheroids, CAMs, and mice.

## 2. Materials and Methods

### 2.1. General Reagents

The following were used in this study: DMEM Glutamax medium (Gibco, Thermo Fisher Scientific, Waltham, MA, USA); gelatin-coated surface; Triton X-100; calcein AM (17783-1MG); ethidium homodimer (EtHD; 46043-1MG-F); MethocelTM (Sigma, St. Louis, MO, USA); fetal bovine serum (FCS; Biowest, Nuaillé, France), penicillin/streptomycin (BioConcept, Allschwil, Switzerland); MycoAlert kit (LT07-218, Lonza, Basel, Switzerland); CellTiter-Glo^®^ for 2D cultures (G7572) and 3D CellTiter-Glo^®^ (G9683) (Promega, Madison, WI, USA); and isoflurane (Aerrane, Baxter Polska Sp. z o. o., Warszawa, Poland).

### 2.2. AGuIX-TPP

AGuIX-TPP is a polysiloxane-based nanoparticle that combines a tetraphenylporphyrin (TPP); a magnetic resonance imaging (MRI) contrast agent, gadolinium (Gd); and a ligand peptide motif (KDKPPR) targeting neuropilin-1 (NRP-1), a receptor overexpressed by angiogenic endothelial cells of the tumor vasculature. AGuIX-TPP was synthesized and provided by the University of Lorraine, as described by Thomas et al. [29]. The nanoparticles were suspended in ultrapure water and NaCl 0.9% (20:80) to obtain an equivalent concentration of 2.5 mM of TPP or 60 mM of Gd freshly before each experiment. For in vitro cell treatment, the AGuIX-TPP concentration range was 0.1–10 μM in the culture medium, and AGuIX-TPP for both the chicken and murine models in vivo was 1.75 μM/kg body weight. These doses were chosen based on previous studies of efficacy and pharmacodynamics [29,34,36,37].

### 2.3. Cell Culture—2D and 3D

ECRF24 immortalized endothelial cells (later referred to as ECRF) were donated by Prof. AW Griffioen. Human glioblastoma multiforme U87-MG cells (later referred to as U87) were generously donated by Prof. M. Barberi-Heyob (Université de Lorraine, CRAN, Vanduvre-lès-Nancy Cedex, France). U87 cells were cultured in a DMEM Glutamax medium (31966-021, Gibco, Brooklyn, NY, USA), ECRF in DMEM/RPMI (1:1) on a 0.2% gelatin-coated surface (G1393-100ML, Sigma Aldrich, Burlington, MA, USA) in a medium supplemented with 10% fetal bovine serum (FCS) (S1810-500, Biowest, Nuaillé, France) and 1% penicillin/streptomycin (4-01F00-H, BioConcept, Allschwil, Switzerland). Cells were cultured at 37 °C in a humidified atmosphere with 5% CO_2_. Cells were monitored for mycoplasma contamination using the MycoAlert kit (LT07-218, Lonza, Basel, Switzerland).

Three-dimensional cultures were established by seeding 1000 cells per well in 96-well U-bottom low-attachment plates (Greiner, Kremsmünster, Austria). The treatment was initiated when the spheroids reached 400 µm in size [38,39]. Spheroid growth was measured using (i) spheroid size and (ii) metabolic activity as an indicator of cell viability. The size of the spheroid was measured with the BioTek Cytation 3 imaging reader (Agilent Technologies, Santa Clara, CA, USA) with the corresponding Gen5 Image software, version 3.04.

### 2.4. Cellular Uptake

A total of 5–10 × 10^3^ ECRF and U87 cells were incubated with AGuIX-TPP (1 μM) for 6 h to 24 h. Then, cells were washed twice with PBS and solubilized in 30 μL of Triton X-100 (Sigma, St. Louis, MO, USA) and 70 μL of a DMSO/ethanol solution (1:3). The accumulation of the photosensitizer was detected by fluorescence measurements (λ_exc_ = 420, λ_em_ = 650 nm) with the microplate reader (Biotek Cytation, Agilent Technologies, Santa Clara, CA, USA).

### 2.5. Illumination of Cells

A total of 5–10 × 10^3^ U87 and ECRF cells were seeded on 96-well plates overnight before the treatment. Thereafter, the medium was removed, and the cells were incubated in a medium containing various concentrations of AGuIX-TPP (0.1–10 μM) for 24 h at 37 °C. Cells were then washed three times with PBS containing calcium and magnesium ions. Subsequently, cells were illuminated through a lid using a home-built diode set emitting 650 nm of red light (18–48 mW/cm^2^) for 5–17 min with corresponding light doses (5, 10, and 20 J/cm^2^). Nonirradiated cells were kept in the dark under similar conditions.

### 2.6. Metabolic Activity Assay

Metabolic activity assays were conducted using the luminescent-based CellTiter-Glo^®^ assay (G7572, Promega, Madison, WI, USA) for 2D cultures and 3D CellTiter-Glo^®^ assay (G9683, Promega, Madison, WI, USA) for 3D cultures, following the manufacturer’s instructions. The tests quantify the ATP that is present, an indicator of metabolically active cells. Luminescence signal intensity was measured with the BioTek Cytation 3 imaging reader, utilizing the Gen5 Image software, version 3.04, with standard settings.

### 2.7. Double Staining of Calcein AM/EtHD

Live–dead staining was performed in 3D cultures using calcein AM and an ethidium homodimer (EtHD). The spheroids were rinsed once with PBS and then stained for 45 min with a solution containing 4 μM of calcein AM and 10 μM of EtHD. Fluorescence images were captured using the BioTek Cytation 3 imaging reader with the Gen5 Image software, version 3.04. Imaging was performed with GFP and Texas Red filter cubes using the 4× and 10× objectives.

### 2.8. PDT of U87 Tumors in the CAM Model

Experiments in the CAM model were performed as described previously [40,41]. Briefly, fertilized chicken eggs (Arare, Geneva, Switzerland) were labeled and placed in a hatching incubator set at 37 °C with 65% relative humidity and an automatic rotator. On day 3 of embryo development (EDD), a 3 mm-diameter hole was made in the eggshell and sealed with Parafilm^®^ (Pechiney, Menasha, WI, USA) to prevent dehydration and infection. The eggs were then returned to the incubator in a static position until needed. On EDD10, the hole above the air pouch was expanded to approximately 3 cm in diameter to allow better access to the chorioallantoic membrane for the experiments. Tumor implantation was performed on EDD10. For spheroid implantation, 25 μL of hanging drops containing 10^6^ U87 cells in 20% Methocel^TM^ (Chempoint, Bellevue, WA, USA) and an 80% serum-free DMEM medium were prepared. Three hours later, the spheroids were transplanted onto the surface of the CAM in the area of implantation that contained a medium-sized vessel, previously slightly disrupted by paper tissue. An alternative method of the implantation of cell suspension was tested, whereby EDD10, a plastic ring, was placed on the surface of the chicken embryo CAMs, and then, 25 μL of cell suspension in a serum-free medium was released within a plastic ring in the area containing a medium vessel, slightly interrupted by paper tissue. Vascularized three-dimensional tumors were visible, measured by a caliper (volume = [larger diameter] × [perpendicular diameter]^2^ × 0.52), and eggs were randomized on EDD10. The formula for the tumor volume used was (v) = abc × π/6; hence, π/6 = 0.52.

AGuIX-TPP (20 J/cm^2^, at drug-light interval (DLI) = 1 min using light at 420 nm for 20 min) was performed on EDD10. When the tumors were treated with PDT, the size of the diaphragm was adjusted so that the irradiation area corresponded exactly to the visible tumor area. Tumor sizes were monitored daily. On the last day of the experiment (EDD14), embryos were sacrificed.

### 2.9. PDT of U87 Tumors in a Murine Ectopic Model

The experiments were carried out according to the European and POLLASA guidelines. Experiment approval was obtained from the II Local Ethics Committee at the Institute of Pharmacology of the Polish Academy of Sciences in Krakow (approval no. 143/2016, 65/2017, 172/2018; dates of approval 31 August 2016, 16 February 2017, and 10 May 2018). Athymic Balb/c nude mice were obtained from Envigo and housed in standard laboratory conditions, with LD: 12/12, humidity: 60%, and temperature: 23 °C, with unlimited access to standard food and drinking water. Mice that were 8–12 weeks old were injected subcutaneously in the right hind leg with 10^6^ U87 cells in 25 μL of PBS. The tumor growth was monitored by a caliper every 24 h, and the volume was calculated from the abovementioned formula.

The antitumor efficacy of AGuIX-TPP photodynamic therapy was evaluated in Balb/c nude mice presenting U87 tumors. Tumor selection for the experiment was performed when the tumor implants reached a size of 30–70 mm^3^, and then, the animals were randomly assigned to one of the following groups: (1) untreated control, (2) light control, and (3) PDT with AGuIX-TPP. The nanoparticle solution was administered by tail vein injection with a dose of PS 1,75 μM/kg body weight. Tumor irradiation was performed at DLI = 15 min using a laser equipped with optical fiber at 650 nm for 17 min, with a laser power of 130 mW/cm^2^, giving a dose of 133 J/cm^2^. The illuminated area, with a diameter of 1.2 cm, was kept constant during irradiation.

### 2.10. Power Doppler Imaging

The High-Resolution Ultrasound Imaging System for small experimental animals (Visual Sonics Vevo 2100, Toronto, ON, Canada) with an MS-550D serial transducer was used. During the procedure, the animal was kept under volatile anesthesia. Anesthesia was induced with 3 vol% isoflurane (Aerrane) and then maintained at 1.5 to 2.0 vol% isoflurane in the air, administered at 1.2 L/min. The mouse temperature was maintained at 37 °C, thanks to the heating bed. To confirm tumor positioning, a B-mode ultrasound (greyscale) was performed at 40 MHz, and vascularization was imaged using Power Doppler ultrasound at 32 MHz with a pulse repetition frequency of 3–4 kHz. Two-dimensional tumor sections with a 12 × 12 mm field of view were captured along the tumor’s third dimension, creating a 3D image. The in-plane resolution was up to 15 μm, with a Z resolution of 200 μm. Tumor and vascular volumes were calculated based on pixel sums from the image slices, with the tumor area manually marked.

### 2.11. EPR Oximetry In Vivo

Measurements of oxygenation were performed with the use of an electron paramagnetic resonance (EPR, Bruker 540L, Bruker, Billerica, MA, USA) and an OxyChip oximetric probe (a generous gift from Dr. P. Kuppusamy and Dr. Maciej Kmiec, Dartmouth Medical School). During the procedure, the animal was kept under volatile anesthesia. Anesthesia was induced with 3 vol% isoflurane (Aerrane) and then maintained at 1.5 to 2.0 vol% isoflurane in the air, administered at 1.2 L/min. The mouse temperature was maintained at 37 °C. thanks to the heating bed. The OxyChip was implanted directly into the tumor with an 18-G needle at least two days before the measurements. After each implantation, a control measurement was performed to ensure the intratumoral placement of the probe and the subsequent observation of spectral changes, i.e., widening or narrowing, depending on the oxygen present around the probe. A spectrometer was used at the L-band (1.2 GHz) with a surface coil for signal detection. The following parameters were set: frequency: 1.2 GHz, power: 6.325 mW, modulation amplitude: 0.18 G, modulation frequency: 100 kHz, sweep width: 5–10 G, sweep time: 10 s, and number of scans: 5–10. After the oximetry, the animal was placed in its cage and monitored till full recovery from the procedure.

### 2.12. RNA Isolation and PCR

RNA isolated from the tumor tissues of U87 ectopic tumors was extracted using the RNeasy Midi Kit (Qiagen, Venlo, The Netherlands). After the extraction, Nuclease-Free Water (AM9938, Ambion, Austin, TX, USA) was added to the eluted samples. RNA quantity was determined using a Nano Drop ND1000 spectrophotometer (Thermo Fisher Scientific, Waltham, MA, USA). The total RNA was transcribed using an iScript cDNA synthesis kit (1708891, Bio-Rad, Hercules, CA, USA), and the mRNA expression of VEGFR2, VEGFA, ANG1, and ANG2 was quantified relative to HPRT by real-time qPCR, in duplicate reactions per sample, with 0.25 μM of forward and reverse primers using the KAPA SYBR FAST qPCR Kit (KK4608, Kapa Biosystems, Wilmington, MA, USA). The primer sequences were as follows: VEGFR2: Forward (5′-3′)-ACAGACCCGGCCAAACAA, Reverse (3′-5′)-TTCCCCCCTGGAAATCCTC; VEGFA: Forward (5′-3′)-AAGGAGAGCAGAAGTCCCATGA, Reverse (3′-5′)-CTCAATTGGACGGCAGTAGCT; ANG1: Forward (5′-3′)-CGAGCCTACTCACAGTACGA, Reverse (3′-5′)-TCGAACCACCAACCTCCTGTT; ANG2: Forward (5′-3′)-GGTGAAGAGTCCAACTACAGGATT, Reverse (3′-5′)-GTTGGAAGGACCACATGCGT; and HPRT: Forward (5′-3′)-CAAACTTTGCTTTCCCTGGT, Reverse (3′-5′)-TCGAGAGGTCCTTTTCACC.

### 2.13. Statistical Analysis

All data were analyzed by two-way ANOVA with Tukey’s HSD post-hoc multiple comparison tests using the GraphPad Prism software (version 9.0.0, GraphPad Software, La Jolla, CA, USA). The number of independent experiments and replicates for each experiment is indicated in the figure legends. The number of animals in each group was selected based on preliminary tumor growth and oximetric experiments with a consideration of the reduction rule of animal research. Differences considered significant are marked with an asterisk in the figures and are described in the legends.

## 3. Results

### 3.1. Accumulation of AGuIX-TPP Within Endothelial and Cancer Cells

**Figure 2:**
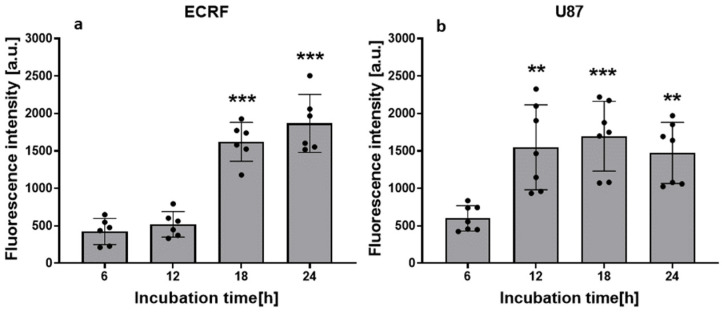
AGuIX-TPP uptake by ECRF and U87 cancer cells. The cells were incubated with 5 μM of AGuIX-TPP for 6 h up to 24 h. The fluorescence intensity of cellular extracts from (**a**) ECRF cells and (**b**) U87 glioma cells; *n* = 2 biological repetitions, in triplicate. ** p < 0.01 and *** < *p* 0.001 vs. 6 h.

To determine the kinetics and maximum uptake of AGuIX-TPP by ECRF endothelial cells and U87 glioma cells, the fluorescence intensity of cell extracts was measured (Figure 2).

The endothelial cells (Figure 2a) showed a similar level of accumulation of the test compound compared to the glioma cells (Figure 2b); however, the uptake was slightly slower than in U87 cells, reaching a high level at 18 h. An incubation time of 24 h was chosen for further in vitro experiments.

### 3.2. Photodynamic Activity of AGuIX-TPP in Endothelial and Glioblastoma Cells In Vitro

**Figure 3:**
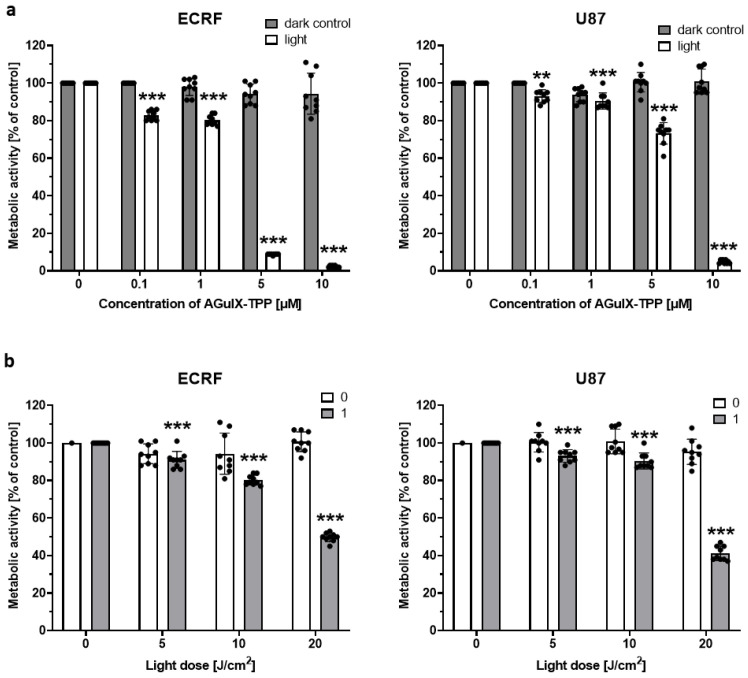
AGuIX-TPP, combined with irradiation, reduced the metabolic activity of ECRF vascular endothelial cells and U87 glioma cells 24 h after treatment. (**a**) Cells incubated with AGuIX-TPP (0–10 μM) and kept in the dark (gray bars) or irradiated with 10 J/cm^2^ (white bars); *n* = 9. (**b**) Cells incubated in the absence of (white bars; 0) or with 1 μM of AGuIX-TPP (gray bars; 1) and irradiated with light doses of 5, 10, and 20 J/cm^2^; *n* = 9. Statistically significant difference, ** p < 0.01 and *** *p* < 0.001; vs. control (dark control) in (**a**) and vs. control (light alone) (**b**). *n* = 3 biological repetitions, in triplicate.

The photodynamic activity of AGuIX-TPP was studied using the metabolic activity of ECRF and U87 cells incubated with the photosensitizer at increasing concentrations in combination with 10 J/cm^2^ irradiation. Figure 3a shows that below 1 μM, AGuIX-TPP exerted no phototoxic effect in any of the cells tested.

**Figure 4:**
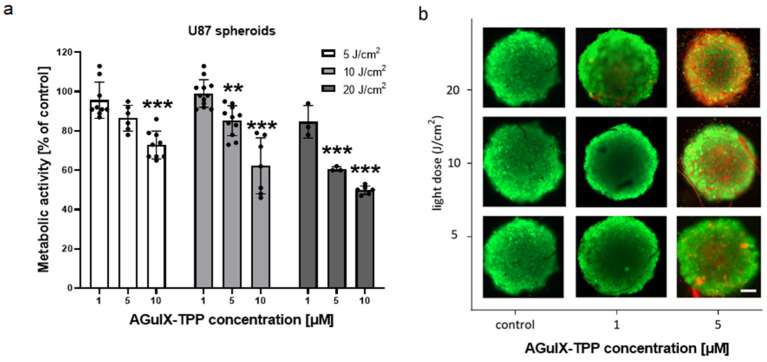
Photodynamic effect with AGuIX-TPP in a 3D culture of U87 cells 24 h after treatment with 1, 5, and 10 μM of AGuIX-TPP and exposure to 650 nm light with a dose of 5–20 J/cm^2^. (**a**) Metabolic activity; *n* = 3–9; ** *p* < 0.01 and *** *p* < 0.001 vs. the lowest concentration. (**b**) Representative fluorescent images of U87 spheroids after PDE; cells stained with calcein AM (green) and EtHD (red). The scalebar represents 200 μm.

In contrast, at a concentration of 1 μM of the photosensitizer and with increasing light doses, ranging from 1 to 20 J/cm^2^, both cell types showed similar sensitivity to treatment (Figure 3b). However, the photosensitizer at a concentration of 5 μM was more effective in the ECRF cells compared to the U87 cells. In both cell types, a light dose of 20 J/cm^2^ effectively reduced metabolic activity by approximately 50%. Since 3-dimensional (3D) spheroids better resemble in vivo conditions, we tested the photodynamic effect in a 3D culture in vitro using U87 glioma cells. Firstly, we optimized the method to obtain reproducible 3D spheroids (Supplementary Figure S1). After this, U87 spheroids of approx. 400 µm were treated with AGuIX-TPP (1–10 μM) and light (5–20 J/cm^2^), and the results are presented in Figure 4. As expected, AGuIX-TPP reduces metabolic activity in a dose-dependent manner (Figure 4a).

Figure 4b shows an increase in the number of dead cells (red) compared to living cells (green) after therapy at 5 μM with a light dose of 5 J/cm^2^, a moderate response with 10 J/cm^2^, and a strong response in cell viability after irradiation with light of a dose of 20 J/cm^2^. However, the use of spheroids resulted in a much lower decrease in cell metabolism (up to 30%) compared to 2D-cultured glioblastoma cells (up to 90%).

### 3.3. PDT with AGuIX-TPP Inhibited Glioblastoma in CAM Model

**Figure 5:**
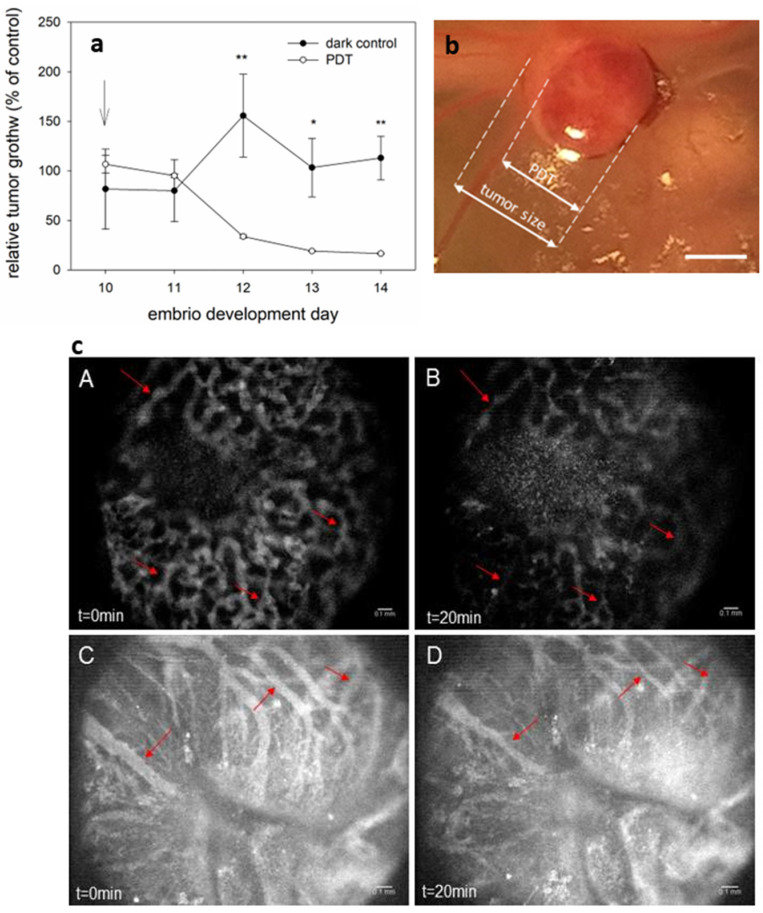
AGuIX-TPP photodynamic therapy inhibits U87 tumor growth in CAMs. (**a**) U87 tumor growth after PDT or dark control (only an injection of 1.75 μM of AGuIX-TPP/kg b.w.) in relation to untreated control tumor growing on the CAM. This graph represents the mean of two independent experiments with SEM. Statistically significant differences between the growth of control and PDT-treated tumors: * p < 0.05 and ** *p* < 0.01. (**b**) Photograph of the U87 tumor on the CAM 24 h after PDT. Scale bar represents 1 mm. (**c**) Fluorescence angiograms taken before (**A**,**C**) and immediately after PDT (**B**,**D**). The vasculature is visualized by FITC-dextran fluorescence angiography (25 mg/kg b.w., 20 kDa, λ_ex_ = 470 nm, λ_em_ > 520 nm). Red arrows show the changes after PDT in the blood vessels and blood stasis. Scale bar represents (**b**) 100 μm and (**c**) 0.1 mm.

A CAM is naturally immunodeficient and highly vascularized, making it an ideal system for tumor implantation and an observation of its growth and vascularity [41,42,43]. Therefore, we investigated PDT with AGuIX-TPP in the tumors growing on the CAM. For this purpose, we performed tumor implantation on embryo development day 10 (EDD10, Supplementary Figure S2), followed by an intravascular injection of AGuIX-TPP (1.75 μM) and an illumination of the tumors with blue light (420 nm, 20 J/cm^2^). Since in the CAM model, the light acts directly on the tumor and the penetration depth is much lower, we used blue light, with an excitation wavelength of 420 ± 20 nm. As shown in Figure 5, the AGuIX-TPP-based PDT effectively decreased tumor growth and caused functional and morphological changes in the vasculature within the tumor. The tumors of the control groups were exposed to AGuIX-TPP alone, injected i.v., which did not decrease the tumor growth ratio (Figure 5a).

Directly after the therapy, necrotic spots were observed macroscopically in the irradiated area (Figure 5b), as well as in blood stasis (Figure 5c, A–D).

### 3.4. Glioblastoma Tumors’ Response to PDT with AGuIX-TPP Depends on Vasculature

**Figure 6:**
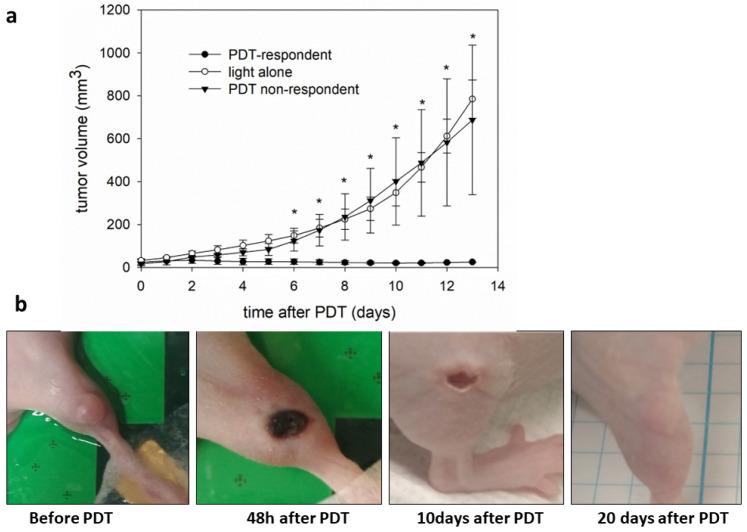
AGuIX-TPP photodynamic therapy inhibits U87 tumor growth. Mice with U87 tumors (approximately 100 mm^3^) were injected with 1.75 µM of AGuIX-TPP/kg b.w. and exposed for 10–17 min to 650 nm of light (130–151 mW/cm^2^). (**a**) Growth curves of tumors after PDT (*n* = 7) or control treatment (light alone); *n* = 2, shown as a mean with SEM, * *p* < 0.05. (**b**) Representative photographs of tumors in the leg after the indicated time.

Figure 6 shows the results of AGuIX-TPP-based PDT in U87 tumors grown ectopically in murine legs. Light alone did not cause significant changes in the tumor growth (Figure 6a). Interestingly, the animals after PDT, showed a dual reaction: the tumors either responded to the therapy or did not change their volume. Tumor growth was inhibited in 48% (10/21) of the mice vs. light control, and 52% (11/21) of the PDT-treated mice did not respond to the therapy at all. Most of the responding mice had tumors under local control for 12 days. Necrosis, scabbing, and transient edema were observed in responding animals (Figure 6b).

**Figure 7:**
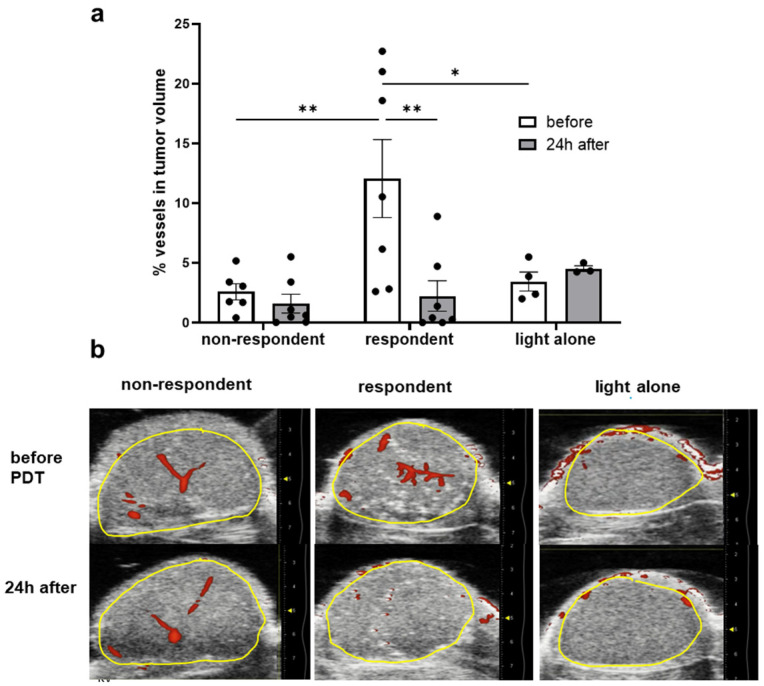
Vascular disruption after PDT with AGuIX-TPP in human glioblastoma tumors in an ectopic mouse model. (**a**) The graphs show the percentage of vessels in the tumor volume, as determined by the US, after PDT (*n* = 14) and light control (*n* = 5), i.e., *n* = 7 for the PDT responders (2 cured, 5 partially responded), *n* = 7 for the PDT non-responders, and *n* = 4–5 for the light control. A statistically significant difference was noted in responding tumors with respect to % vessels in tumor volume before and 24 h after therapy; * *p* < 0.05, ** *p* < 0.01. (**b**) Representative single-slice images of ectopic U87 tumors, before and after PDT therapy, were acquired with Power Doppler, showing the vasculature with blood flow (red) and the tumor area (yellow line). The original scale bar on the left side of each image shows that the tumors were between 2 and 7 mm deep. Yellow dot marks a 5 mm distance from the transducer.

To analyze the vascular functionality of the tumor and surrounding tissues, we used the Power Doppler ultrasound (Figure 7). The influence of PDT on the vascular system of the tumor was evaluated by calculating changes in the percentage of visible, i.e., functional, blood vessels in the tumor volume. The images seen in Figure 7b represent a single slice, showing varied amounts of vasculature in the tumors. It is important to note that the percentage of vessels was determined from the whole tumor volume (multiple slices per tumor).

The vascular structure in the untreated U87 tumors was very heterogeneous not only within a single tumor but also between tumors. As seen in Figure 7, the initial vessel volume, expressed as a percentage of the tumor volume, varied from 0 to 22%. There was a visible trend of non-responding tumors, characterized by a low initial vascular percentage, compared to partially responding and cured mice. In future studies, as the vasculature density and resulting tumor pO_2_ are factors determining the PDT response, at least one of these parameters should be determined before the treatment, and animal groups should be randomized based on vascular density.

**Figure 8:**
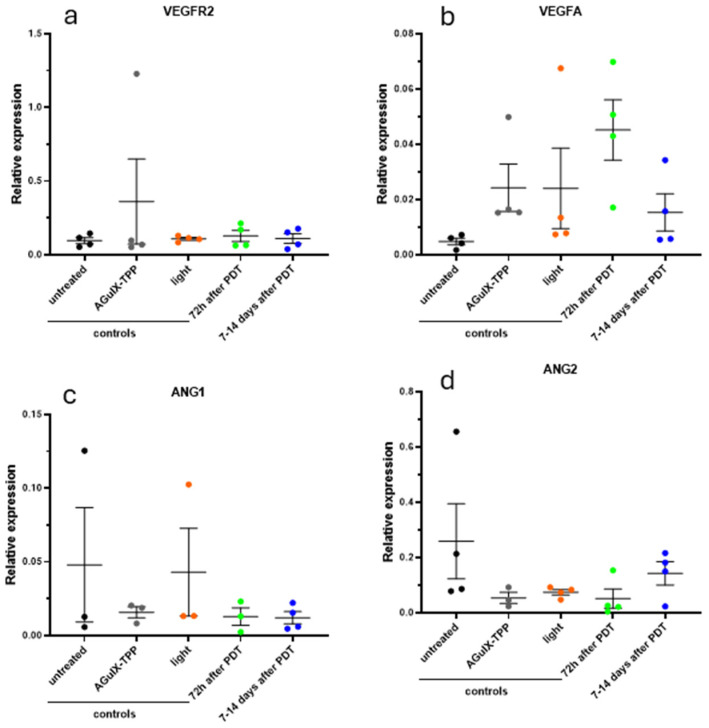
Expression of VEGFR2, VEGFA, ANG1, and ANG2 mRNA in the ectopic U87 tumors. Real-time qPCR of VEGFR2 (**a**), VEGFA (**b**), ANG1 (**c**), and ANG2 (**d**), showing mRNA levels in the ectopic U87 tumors (*n* = 3–4 mice/group). Values represent fold changes relative to HPRT mRNA levels.

Next, we investigated how PDT affected the expression of VEGF and angiopoietins, using qRT-PCR. Two endothelial-specific growth factors, Angiopoietin-1 (Ang1) and Angiopoietin-2 (Ang2), have been shown to interact with VEGF to determine the fate of blood vessels during angiogenesis. Although our data indicate an increase in VEGFA expression after 72 h (Figure 8), due to high heterogeneity, this change was not statistically significant, while angiopoietin levels did not change. The VEGFR2 expression was relatively stable across individuals.

**Figure 9:**
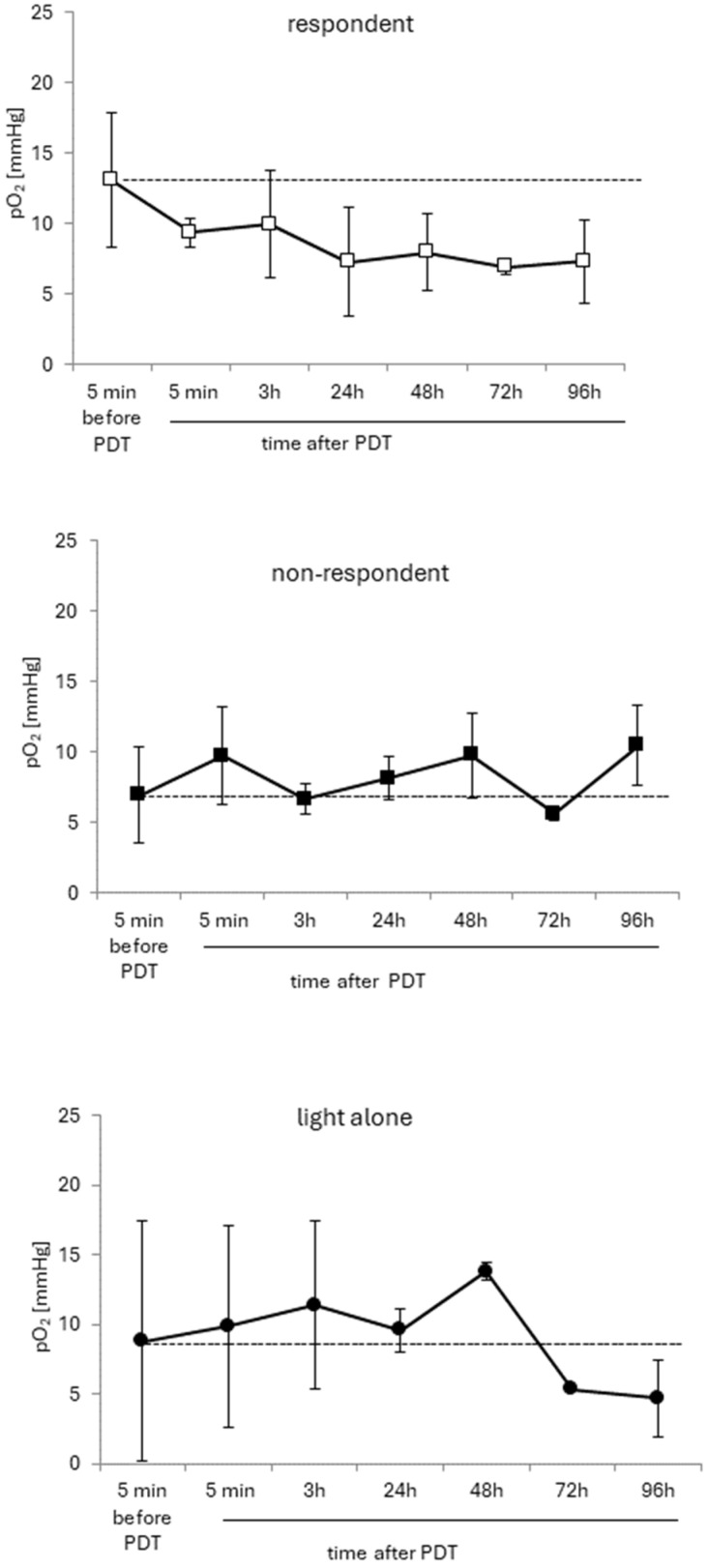
Changes in oxygenation in the ectopic U87 tumors after PDT with AGuIX-TPP. The graphs show the mean pO_2_ in tumors at the indicated time points, before and after PDT; *n* = 3 for the group responding to PDT, *n* = 4 for non-responders to PDT, and *n* = 2 for the light control group.

Oximetry in tumors in vivo was performed to link the observed changes in the tumor vascular system with changes in the tumor pO_2_. The EPR in vivo measurement technique with the OxyChip oximetric probe enables the local measurement of the partial pressure of oxygen. The basic median of the tumor pO_2_ before the treatment was diverse and was between 5 and 15 (+/−10) mmHg. After the treatment in all mice, the pO_2_ oscillated around the initial value. The initial pO_2_ value might have been slightly lower in non-responding mice, although this was not statistically significant (Figure 9). However, a steady decrease in pO_2_ with time after PDT was observed in the group of responders (Figure 9 upper), in contrast to the other groups, non-responders and light control (Figure 9 middle and bottom), where the pO_2_ values remained close to the initial pO_2_ value.

## 4. Discussion

AGuIX nanoparticles were first synthesized by Prof. Tillement’s team for MRI-guided radiotherapy and were designed to improve tumor targeting and radiosensitization [44]. AGuIX nanoparticles have demonstrated high radiosensitizing properties and excellent MRI-positive contrast properties due to the paramagnetic properties of gadolinium [45].

The platform AGuIX may be combined with different metal ions and ligands. For example, the combination of AGuIX with Terbium (Tb) as a nano scintillator has been shown to facilitate energy transfer to the photosensitizer, resulting in increased reactive species production and improved cell-growth arrest in glioblastoma cells under X-ray exposure [35]. The nanoparticles also exhibit radiosensitizing properties by exacerbating radiation-induced DNA damage and reducing DNA repair, as observed in non-small-cell lung cancer and triple-negative breast cancer models, thereby enhancing the overall therapeutic efficacy [46,47]. Furthermore, AGuIX nanoparticles labeled with ^89^Zr for PET imaging have demonstrated efficient tumor infiltration and prolonged retention in glioblastoma models, indicating their potential for precise tumor targeting and treatment monitoring [46]. Nanoparticles containing Gd ions (no photosensitizer) undergo several clinical trials for radiochemotherapy in the treatment of brain metastases, lung tumors, pancreatic cancers, and advanced cervical cancer. Another clinical study is in phase II of a clinical test for radiochemotherapy and concomitant temozolomide in patients with newly diagnosed glioblastoma [48]. There have been several attempts to apply PDT treatment to brain tumors [49,50,51]. A main obstacle is the inflammation resulting from tissue destruction, leading to edema. This has been proposed to be resolved by anti-inflammatory drugs. The results of PDT in the intraoperative interstitial treatment of glioma patients are encouraging [52].

AGuIX nanoparticles have shown significant promise in enhancing the efficiency of photodynamic therapy (PDT) for tumor treatment through various mechanisms. For instance, AGuIX nanoparticles functionalized with a porphyrin photosensitizer and a KDKPPR peptide ligand targeting neuropilin-1 (NRP-1) have demonstrated an increased uptake by M2 macrophages and human umbilical vein endothelial cells (HUVECs), leading to enhanced PDT effects and tumor vessel destruction [29,53]. The pros of AGuIX over other nanocarriers are that the coupled drug is already exposed and does not have to be transported out of the cell, which is potentially important for therapeutic outcomes [29]. AGuIX was also shown to pass the blood–brain barrier and, due to selective binding, does not damage normal tissue upon light exposure [29]. Overall, the multifaceted approach of AGuIX nanoparticles, including their ability to target tumor vasculature, enhance radiosensitivity and improve imaging capabilities, providing strong evidence for their efficiency in the photodynamic therapy of tumors [34].

AGuIX nanoparticles, coupled with a photosensitizer and peptide, were described to have an average diameter of 10 nm [29], so their size should allow cells to uptake them from the cell culture medium. In the present study, cells were shown to effectively accumulate nanoparticles at 1 μM. In previous work, Panc1 pancreatic cancer cells were shown to capture AGuIX (of a size of approx. 5 nm) by endosomal vesicles and transport into the cytoplasm [54].

The in vitro results show that the tested compound does not have toxic effects in the dark. These results are consistent with a study by Thomas and Gries, showing that AGuIX-TPP and both peptides are non-toxic. It has also been described that the platform itself is biocompatible and non-toxic [54]. PDE was effective for both the EC and GBM cell lines. Studies by Thomas et al. using the same compound in HUVECs, showed effective PDE with AGuIX-TPP [29].

In spheroids, despite worse penetration, the effects are seen inside the sphere; however, the higher concentrations of 5 and 10 µM and the same light doses of 10 and 20 J/cm^2^ were used, and metabolic activity did decrease only to 50% and not lower. Higher resistance in spheroids is usual, due to cell-to-cell contacts [55]. The spheroid architecture is crucial to understanding and explaining the changing environment at the depth of the tumor. The rim of the spheroid is more exposed to a drug-enabling uptake, whereas the core of the spheroid is more compact, and the tight junctions between cells are closer. Furthermore, the oxygen concentration and availability of cells vary through the layers of the spheroid, and the core of the spheroid can be hypoxic and often already necrotic [56]. These factors may influence the lower response to PDT.

The immediate vascular effect after PDT with AGuIX-TPP was estimated in the CAM model. PDT led to blocking the vasculature and inhibition of tumor growth in the CAM model (Figure 5). These findings align with results observed in mouse studies. However, the short observation time (4 days) prevented studying longer-term effects and tumor regrowth.

In U87, the GBM tumors that grew in the legs of the nude mice, approximately in half of the mice the tumor growth was inhibited, and the other half did not show any response. In the responding animals, a part of the tumors regrew after therapy. In an orthotopic model of glioblastoma in nude rodents, two similar groups, responding and non-responding to therapy, were observed [36,37,57]. Our previous studies have shown that moderate hypoxia after PDT does not lead to a positive therapeutic outcome. In contrast, it was shown that very strong hypoxia due to PDT leads to a cure [58,59]. Medium oxygen levels, measured in GBM tumors in the present study, varied from 5 to 15 mm Hg. These are typical values for many tumor types in mice. High intratumoral pO_2_ heterogeneity was described using 3D tumor oxygen mapping [60]. After the treatment, the pO_2_ did not decrease completely but remained around 5 mm Hg, suggesting that this level of hypoxia might be stimulating tumor regrowth, as mentioned above [59,61].

The initial level of vascularity and its functionality correlated with therapeutic outcomes (Figure 6 and Figure 7). Tumors with low levels of vascular functionality during therapy (measured before therapy) showed a worse therapeutic outcome. However, the vascularity of the U87 tumors was very heterogeneous. The percentage of vessels was determined from the whole tumor volume (multiple slices per tumor), providing more comprehensive data. Of note is the high variance of the vascularity in the group of responding tumors before treatment, which dropped significantly after the treatment, demonstrating its efficacy. In contrast, CAM tumors were uniformly highly vascularized. The greater vascularity in the CAM model contributes to more consistent outcomes of PDT. Our results underline the importance of assessing the functionality of the vasculature in the individual tumors when considering therapies aimed at this compartment.

Impairment of both the structure and function of tumor vessels impacts a tumor’s microenvironment [62]. It can deepen already-present hypoxia and nutrient deprivation, limit the infiltration of the immune cells, facilitate the extravasation of tumor cells, and limit the penetration of chemotherapeutics.

A possible explanation for tumor regrowth is that the light distribution during PDT was not efficient. The light wavelength used was 650 nm, and the penetration of this wavelength is only 1–2 mm [63]. Irradiated tumors could be too large and/or too compact and light impermeable. The distribution of photosensitizers could be uneven in some tumor compartments, which can lead to inadequate levels of oxidative stress in cells and uneven PDE. Oxygenation is also distributed unevenly [64], so the efficacy of PDT after irradiation after PS injection can be limited. The heterogeneity of the tumor itself can also affect the effective dose of light and drugs and the uneven distribution of oxygen. The DLI used in this study was designed to allow irradiation of the compound present in blood vessels (1 min in CAMs, 15 min in mice). Studies by Thomas, Gries, and Toussaint [29,36,37] showed that the photosensitizer remains in the vessels/endothelial cells for up to 4 h. By choosing the 15 min DLI, we ensured the antivascular nature of the therapy. Importantly, the decrease in vascular functionality after the applied therapy was confirmed in the responding mice before and after treatment by ultrasound. GBM, in comparison to other tumors, is characterized by being well-vascularized with highly abnormal blood vessels. These vessels often have multiple layers of endothelial cells, show uncontrolled proliferation, and are prone to leakage, among other irregularities [65]. This could also contribute to heterogeneity. As seen in Figure 7, the percentage of vasculature in the tumor volume is heterogeneous, rising to 30%. It was shown that in the responding tumors, the blood vessels were completely closed 24 h after therapy, while in the partially responding mice, the blood vessels did not close 24 h after treatment. Previous AGuIX-TPP studies show efficacy, in mice and rats and in an orthotopic model with different DLIs [45]. Another limitation of this study was the light penetration through the tissue. Using photosensitizers, absorbing light in the 750–800 nm range would improve the penetration depth in the tissue. On the other hand, ensuring sufficient oxygen supply in the tumor, e.g., by using oxygen nanobubbles [66], might lead to increased efficacy. However, the side effects might include an increased metastatic potential and increased normal tissue damage. As has been shown by Gries et al., PDT using AGuIX-TPP in the brain might be limited by an inflammatory response [36].

There have been several attempts to apply the PDT treatment to brain tumors [49,51,67]. One main obstacle is the inflammation resulting from tissue destruction, leading to edema. This has been proposed to be resolved by anti-inflammatory drugs. The results of PDT in the intraoperative interstitial treatment of glioma patients are encouraging [51,52,68,69].

Another limitation of our study is that using nude mice eliminates interactions with the immune system. It would be useful to study the effects of PDT with AGuIX on the vascular system in the presence of a fully functioning immune system to fully comprehend the complex interactions in the tumor microenvironment after treatment.

In summary, PDT using AGuIX-TPP was effective against glioma in both cells and spheroids. It shows high efficiency in damaging vasculature in CAMs, whereas in animal tumors, its effectiveness is strongly related to the presence of vasculature within the tumor and achieving tissue anoxia after treatment. This was not fully accomplished in the present study. The success of vascular-targeted PDT requires two conditions: (i) the presence of functional vessels in the tumor before treatment and (ii) the effective blocking of this vasculature after treatment, leading to a complete decrease in pO_2_.

## 5. Conclusions

The rich vascularization of a tumor at the initiation of therapy is an important and key element that determines the success of the therapy. Although tumors showing strong vascularization are good candidates for treatment with vascular-targeted PDT using AGuIX-TPP, this therapy needs further research and refinement to effectively and reliably lead to a state of tissue hypoxia after treatment, which is a key factor affecting treatment efficiency.

## Data Availability

Data are available upon request.

## Supplementary Materials

The following supporting information can be downloaded at: https://www.mdpi.com/article/10.3390/cancers16233924/s1; Figure S1: Optimization of the 3D spheroid model of cultured glioblastoma U87 cells. Spheroid kinetics and morphology of U87; Figure S2: Optimization of the chicken CAM model of U87 human glioblastoma tumors.
