# Supplementary material for: Preclinical Photodynamic Therapy Targeting Blood Vessels with AGuIX® Theranostic Nanoparticles
Source: Cancers (Basel). 2024 Nov 23;16(23):3924. doi: 10.3390/cancers16233924 (PMC11640380; doi:10.3390/cancers16233924)
Supplement: Supplementary file 1 [file cancers-16-03924-s001.zip › cancers-3315936-supplementary.pdf]

cancers MDPI

Supplementary materials

Preclinical Photodynamic Therapy Targeting Blood Vessels with AGuIX® Theranostic Nanoparticles

Ewa Kowolik [1], Dariusz Szczygieł [1], Małgorzata Szczygieł [1], Agnieszka Drzał [1], Kalyani Vemuri [2], Anna-Karin Olsson [2], Arjan W. Griffioen [3], Patrycja Nowak-Sliwinska [4,5,6,*], Agnieszka Wolnicka-Glubisz [1] and Martyna Elas [1,*]

[1] Department of Biophysics and Cancer Biology, Faculty of Biochemistry, Biophysics and Biotechnology, Jagiellonian University, 7 Gronostajowa Street, 31-387 Krakow, Poland; ewa.kusber@gmail.com (E.K.); dariusz.szczygiel@uj.edu.pl (D.S.); gosia.szczygiel@uj.edu.pl (M.S.); agnieszka.drzal@uj.edu.pl (A.D.); a.wolnicka-glubisz@uj.edu.pl (A.W.-G.)
[2] Department of Medical Biochemistry and Microbiology, Biomedical Center, Uppsala University, Husargatan 3, SE-75123 Uppsala, Sweden; kalyani.vemuri@imbim.uu.se (K.V.); anna-karin.olsson@imbim.uu.se (A.-K.O.)
[3] Angiogenesis Laboratory, Department of Medical Oncology, Amsterdam UMC, Cancer Center Amsterdam, 1081 HV Amsterdam, The Netherlands; a.griffioen@amsterdamumc.nl
[4] School of Pharmaceutical Sciences, University of Geneva, 1211 Geneva, Switzerland
[5] Institute of Pharmaceutical Sciences of Western Switzerland, University of Geneva, 1211 Geneva, Switzerland
[6] Translational Research Center in Oncohaematology, 1211 Geneva, Switzerland
* Correspondence: patrycja.nowak-sliwinska@unige.ch (P.N.-S.); martyna.elas@uj.edu.pl (M.E.); Tel.: +41-22-379-3352 (P.N.-S.); +48-12-664-338 (M.E.)

To test the photodynamic effect in the 3D *in vitro* glioblastoma model, human U87 glioblastoma cells were used to form reproducible 3D spheroids. The seeding density was optimized at 1000 to 1500 cells/well to obtain spheroids with a diameter of 500 µm on day 3 after seeding. The spheroids were maintained for ten days. As measured by diameter, spheroids started with 400 reaching 1100 µm at day 10. The spheroids formed compact round structures on day 1, which increased in size over time (Figure S1).

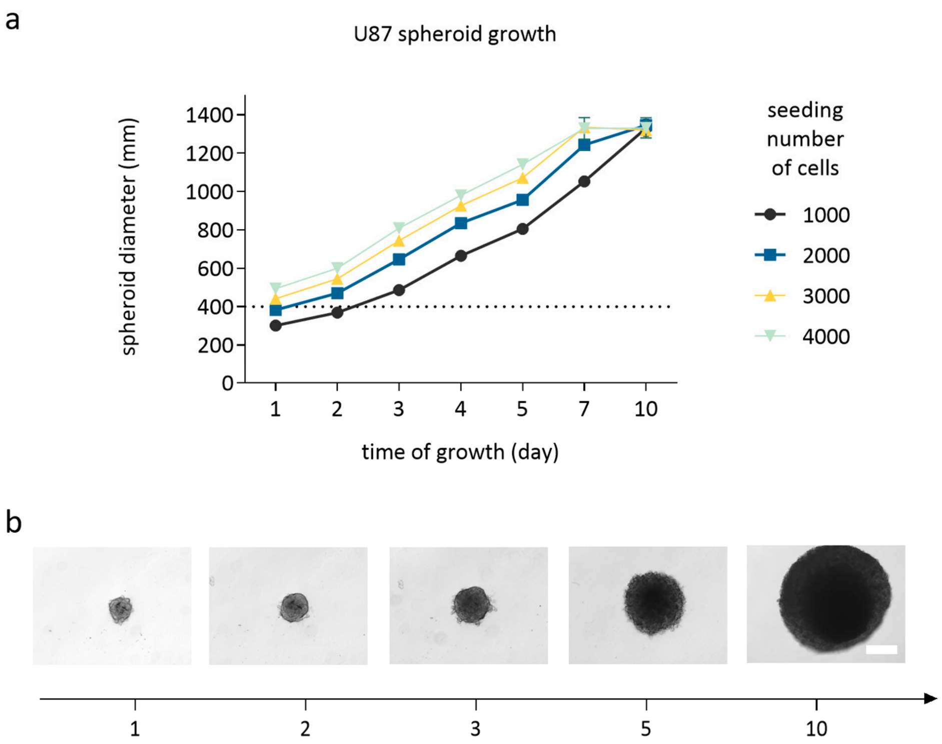

Supplementary Figure S1. Optimization of the 3D spheroid model of cultured glioblastoma U87 cells. Spheroid kinetics and morphology of U87. (**a**) Growth kinetics represented in the diameter of the spheroid over 10 days at a seeding density (1000-4000 cells/well) N=3 (**b**). Representative images of U87 spheroids grown at the optimal seeding density of 1500 cells between days 1 and 10. The scale bar represents 300 μm.

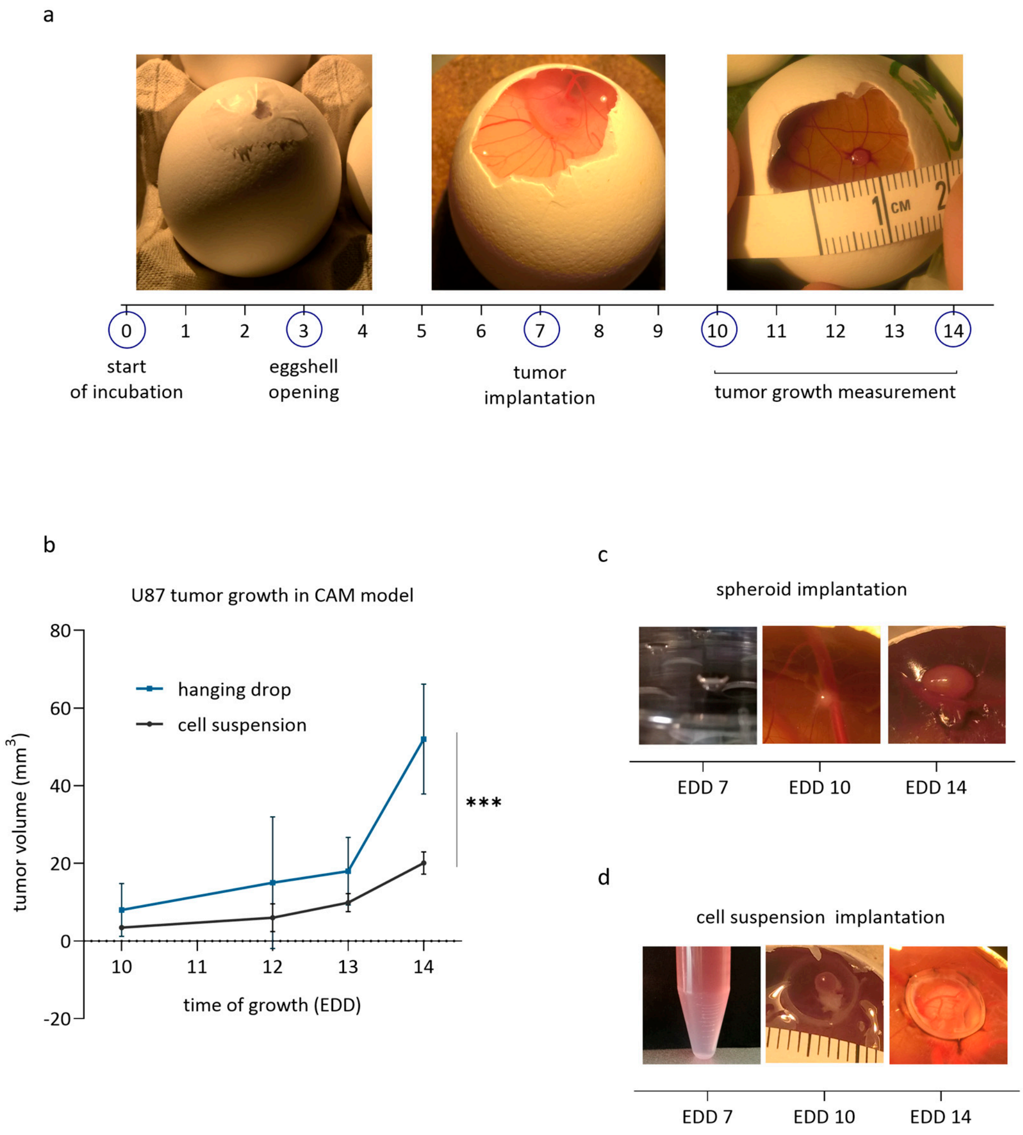

Supplementary Figure S2. Optimization of the chicken CAM model of U87 human glioblastoma tumors. (**a**) Cultivation protocol that presents the time frames for model maintenance. Growth curves of U87 tumors growing in CAM for different implantation methods. (**b**) Representative images of the stages of the hanging drop and cell suspension implantation methods in CAM. Photographs show a model in different stages of cultivation (**c,d**). n=3, ***P<0.001 A statistically significant difference was observed in tumor sizes on day 14 between hanging drop vs cell suspension implantation methods.
